# Age-Related Variations in Clinical Profiles for Children with Sports- and Recreation-Related Concussions

**DOI:** 10.3390/diagnostics14182042

**Published:** 2024-09-14

**Authors:** Sicong Ren, Daniel J. Corwin, Catherine C. McDonald, Daniele Fedonni, Christina L. Master, Kristy B. Arbogast

**Affiliations:** 1 Center for Injury Research and Prevention, The Children’s Hospital of Philadelphia, Philadelphia, PA 19104, USA; corwind@chop.edu (D.J.C.); mcdonalc@nursing.upenn.edu (C.C.M.); fedonnid@chop.edu (D.F.); masterc@chop.edu (C.L.M.); arbogast@chop.edu (K.B.A.); 2 Perelman School of Medicine, University of Pennsylvania, Philadelphia, PA 19104, USA; 3 Division of Emergency Medicine, The Children’s Hospital of Philadelphia, Philadelphia, PA 19104, USA; 4 School of Nursing, University of Pennsylvania, Philadelphia, PA 19104, USA; 5 Sports Medicine and Performance Center, The Children’s Hospital of Philadelphia, Philadelphia, PA 19104, USA

**Keywords:** concussion, mild traumatic brain injury, pediatric, adolescents, children, sports, recreation, clinical profile, symptoms

## Abstract

Objective: The purpose was to examine clinical profiles in concussed children aged 5–9 and 10–12 years and compare them with those of adolescents >12 years. Methods: This study included patients aged 5–18 years presenting to a specialty care concussion program with a sports- and recreation-related (SRR) concussion ≤28 days postinjury. Demographics, injury mechanisms, symptoms, and clinical features were assessed. Chi-squared tests, one-way ANOVA, and Kruskal-Wallis were used for comparisons across age groups. Results: A total of 3280 patients with SRR concussion were included: 5.0% were 5–9 years, 18.4% were 10–12 years, and 76.6% were 13–18 years. Younger age groups had more males than females (5–9 years: 70.7% vs. 29.3%) and more commonly sustained their injury during limited- (28.7%), and non-contact (7.9%) activities compared to other age groups (*p* < 0.01). Younger children presented less symptoms frequently (*p* ≤ 0.042), but higher symptom severity in somatic and emotional domains (*p* ≤ 0.016). Fewer 5–9-year-olds reported changes in school (25.6%), sleep (46.3%), and daily habits (40.9%) than adolescents (*p* < 0.001). Conclusions: Among SRR-concussed children and adolescents, we found significant age-related variations in demographics, injury mechanism, symptoms, and clinical features. Recognizing these unique features in younger children may facilitate targeted management and treatment.

## 1. Introduction

Organized sports are popular recreational activities in the youth population. In 2022, over 60% of youths aged 6–17 years were involved in team sports, with high participation across genders, income, races, and ethnicities [1]. While sport involvement is beneficial across multiple domains of pediatric health and wellness [2], with it comes the risk of injury. Concussion in sports has received increasing attention due to the public awareness of injury by the medical and scientific communities and concern about both potential short- and long-term consequences. Concussions in the pediatric population comprise around 61% of sports- and recreation-related (SRR) injuries [3], and approximately one to two million children aged 0–18 years sustain an SRR concussion in the United States annually [4]. SRR concussions generally result in a constellation of acute and sub-acute deficits across multiple domains (memory, concentration, sleep, processing speed, and visual and motor functions) [5,6,7]. Growing evidence suggests that pediatric SRR concussions may present more challenges in diagnostic and symptom management than adults [8,9], possibly due to ongoing neurologic development [10,11]. 

The most recent International Consensus Conference on Concussion in Sport (2022) recommends an active therapeutic approach [12]; however, this was derived mainly from studies on adolescents. The report in particular calls for a need for understanding more about concussions in younger children, particularly those sustained in sports and recreation [12,13]. Younger children less than 12 years of age are increasingly participating in organized sports [1], where early sports competition and lack of physical readiness during childhood could increase the risk of sports-related injury [14]. Since younger children have a greater head-to-body ratio, and weaker neck muscles than adolescents [15], they may be more vulnerable to concussion, and if injured, variability in physiology across pediatric development may lead to a differing presentation of concussion symptoms. The limited prior studies in younger children indicate differences with age in the relationship between symptom profiles and time to symptom resolution. In those aged 7–12 years, greater somatic and cognitive symptom burden was associated with a longer recovery, while in those aged 13–18 years, vestibular–ocular symptoms appeared to drive a longer symptom recovery [10]. Other work has demonstrated that, even within the 5–11 year age group, visio-vestibular deficits were more prevalent in 9–11-year-olds than their younger counterparts [16]. Additionally, approximately one-third of patients with concussions who were seen in a large regional pediatric health care network were 5–11 years old [17], emphasizing the critical need to understand injury characteristics in this age group. 

Therefore, the purpose of this study was to characterize the demographics, injury mechanisms, initial presentation, and clinical characteristics of SRR concussions among children aged 5–12 years and compare those characteristics with concussed adolescents aged 13–18 years.

## 2. Materials and Methods

### 2.1. Study Design

Data were obtained from the Minds Matter Concussion Registry, which prospectively collects data using electronic health record (EHR) information for patients seen for concussion in multiple settings across a large, regional pediatric care network [18,19,20].

### 2.2. Participants

The population for this analysis was limited to patients with SRR concussions aged 5–18 years, presenting between 1 January 2018 and 4 June 2024, for their initial visit to the network’s specialty care concussion program within 28 days of injury. Exclusion criteria included patients outside the target age range; initial clinical visit was earlier than 1 January 2018; days between injury to initial clinical visit were greater than 28 days; and the mechanism of injury was non-sports and recreation. SRR concussions were defined as injuries occurring during organized and/or competitive sports, recess, physical education class, free play, or sport activity in a noncompetitive environment [21]. Subsequently, SRR concussions were classified by the contact level associated with that activity using a classification system developed by the American Academy of Pediatrics’ Committee on Sports Medicine and Fitness [22]. Patients were diagnosed with a concussion by a sports medicine pediatrician using the definition of concussion set forth in the most recent Consensus Statement on Concussion in Sport at the time [23], including a history of an injury with transmitted forces to the head followed by the onset of symptoms associated with concussion. Parents and patients completed an online questionnaire prior to the first visit that included demographic information, patient/parent-reported medical history, and self-reported symptoms. The study was deemed exempt by the institution’s Institutional Review Board. 

### 2.3. Variables of Interest

Biological sex, age at visit, race, ethnicity, days from injury to visit, days of time in care (i.e., days between initial visit and final visit), and concussion history were collected via the EHR. Furthermore, pre-existing medical conditions that might influence concussion recovery and evaluation were collected via the survey completed by parents prior to the first visit, including learning/developmental problems, mood-related problems, vision/vestibular problems, physical problems, and therapy. Additionally, self-reported (by either patient or parent) changes in daily school and home life were collected.

The mechanism of injury was categorized into standardized injury codes (e.g., fall, struck by object, struck by person). The categories for SRR concussions included contact or collision sports, sports with limited or no contact, and other activities. Other activities were defined as injuries occurring during free play, physical education class, recess, fitness training, and summer camp, or as missing values. In each category, we classified the sports presenting with less than 1% for each age group as “Other”. Activities not included in the American Academy of Pediatrics’ system were assigned categories based on the likelihood of contact or collision in alignment with prior work [16].

Lastly, we examined all patient- or parent-reported (as age appropriate) concussion-related symptoms obtained using the validated and reliable Post-Concussion Symptom Inventory (PCSI) [24], specifically the PCSI-Child for those aged 5–12 years and the PCSI-Teen for those aged 13–18 years. We chose to evaluate the 16 PCSI items common across both PCSI versions, including headaches, nausea, sensitivity to light, sensitivity to noise, dizziness, balance problems, vision problems, difficulty in concentrating, difficulty in remembering, feeling slowed down, mentally foggy, drowsiness, feeling fatigue, irritability, sadness, and nervousness. As each symptom is reported on a 0–2 scale for the PCSI-Child and on a 0–6 scale for the PCSI-Teen, to directly compare PCSI severity scores, each PCSI item score was divided by the maximum item score and then multiplied by 100, yielding the percentage of symptom severity. To compute the item “slowed down” from PCSI-Teen, which is not explicitly asked in PCSI-Child for those aged 5–12 years, we added the score for “moving slowly” and “thinking slowly” together, and thus normalized this item to the maximum 2-item score of 4.

### 2.4. Statistical Analysis

We categorized the children aged 5–12 years into 2 groups, 5–9 years and 10–12 years, since children generally graduate from elementary school at this time, and peri-pubertal changes often begin occurring around age 10. We then compared these groups to the more frequently studied age group of 13–18 years. Descriptive analyses were conducted based on the distribution of demographic and clinical characteristics among patients with SRR concussions. Frequencies and proportions for categorical variables were calculated. For continuous variables, the mean and standard deviation were calculated if data were normally distributed; and median and interquartile range were calculated if data were not. We compared the distribution of these variables by age group using the chi-squared test (for categorical variables) and the Kruskal–Wallis or one-way ANOVA tests (for continuous variables) depending on data distribution. Post hoc tests were conducted using Fisher’s exact tests (for categorical variables), and pairwise Wilcoxon rank-sum or pairwise *t* tests (for continuous variables) with Bonferroni adjusted *p*-values, with a significance level set at 0.05. All statistical analyses were performed using R version 4.4.0 (R Foundation for Statistical Computing, Vienna, Austria).

## 3. Results

### 3.1. Demographic and Clinical Characteristics

**Figure 1:**
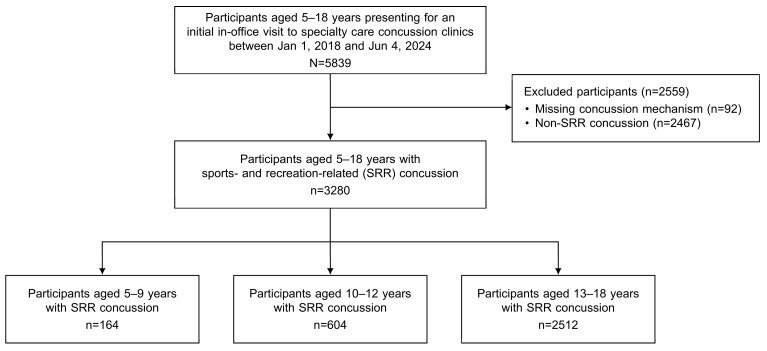
Flowchart of cohort derivation for this study.

A total of 3280 children and adolescents with SRR concussions were included in this study (Figure 1). The demographic characteristics of this sample are described in Table 1. Overall, 46.5% of patients were female and 70.7% identified as non-Hispanic White. Distribution of age was as follows: 5.0% were 5–9 years of age, 18.4% were 10–12 years, and 76.6% were 13–18 years. In the younger age groups, the number of male patients was significantly greater than that of female patients (5–9 years: 70.7% vs. 29.3%, *p* < 0.001; 10–12 years: 61.4% vs. 38.6%, *p* < 0.001) in contrast to the 13–18-year-old patients (50.5% vs. 49.5%, *p* = 0.632). Previous concussions were less common among those in the younger age groups (5–9 years: 14.0% vs. 10–12 years: 30.6% vs. 13–18 years: 42.3%, *p* < 0.001). There was no significant difference in the number of days between the date of injury and initial clinic visit or days of time in care across age groups. Pre-existing medical conditions are shown in Supplementary Table S1. 

### 3.2. Classification of Sports and Recreation Activities

Table 2 presents SRR activities classified by contact level and age group. The majority of SRR concussions occurred during contact or collision sports across all age groups, within a lower proportion in the youngest age group (58.5% in 5–9 years, 72.0% in 10–12 years, and 70.6% in 13–18 years, *p* < 0.001). The top two most common contact or collision sports were soccer and football (in varying order) for each age group, while the third most common was basketball.

For limited-contact sports, concussions in 5–9-year-olds mainly occurred during baseball (5.5%) and skiing (4.3%); concussions in 10–12-year-olds mainly occurred during cheerleading (4.0%) and baseball (3.1%); and concussions in 13–18-year-olds mainly occurred during cheerleading (5.4%), volleyball (3.7%), and softball (3.4%).

For non-contact sports, swimming was identified as the dominant activity leading to concussion across all age groups (5–9 years: 3.7%, 10–12 years: 0.5%, and 13–18 years: 1.1%). The mechanism of concussion sustained while swimming includes being struck by a person, struck by or against objects, and falling. The 5–9-year-olds had a significantly greater proportion of concussions sustained in non-contact sports than the other age groups (5–9 years: 7.9%, 10–12 years: 2.2%, and 13–15 years: 3.1%, *p* < 0.001). The youngest age group (5–9 years) demonstrated a greater proportion of participants (4.9%) sustaining their concussion during other activities, including playing and physical education class, than other age groups (10–12 years: 2.0%, 13–18 years: 0.6%, *p* = 0.347), though the difference did not reach significance.

### 3.3. Symptom Presence and Severity Profile

Symptom presence for common items among age groups is described in Table 3. Overall, as the age increased, the proportion of endorsement of 11 out of 16 symptoms also increased. For example, there were significant age-related differences in the rate of endorsement of headaches (5–9 years: 78.4% vs. 10–12 years: 82.5% vs. 13–18 years: 86.0%, *p* = 0.007), balance (28.9% vs. 39.6% vs. 50.5%, *p* < 0.001), and difficulty in concentrating (52.3% vs. 59.9% vs. 73.8%, *p* < 0.001). However, no significant differences were observed in the rates of endorsement of nausea, feeling slowed down, drowsiness, irritability, and nervousness across age groups (*p* = 0.050–0.627).

Normalized symptom severity scores for common items among age groups are described in Table 4. Generally, younger age groups demonstrated greater severity scores in somatic and emotional symptoms, but lower severity scores in cognitive symptoms than 13–18-year-olds. For example, younger age groups had significantly higher severity scores for headaches (5–9 years: 52.6% vs. 10–12 years: 59.4% vs. 13–18 years: 47.8%, *p* < 0.001) and sadness (18.6% vs. 16.2% vs. 13.6%, *p* = 0.016), but significantly lower severity scores for difficulty in concentrating (30.4% vs. 39.9% vs. 40.3%, *p* = 0.002). Patients aged 10–12 years demonstrated greater severity scores for drowsiness (45.8% vs. 33.5%, 31.8%, *p* < 0.001) and fatigue (46.1% vs. 35.5%, 37.5%, *p* < 0.01) than 5–9- and 13–18-year-olds.

### 3.4. Change in Daily Activities

Table 5 shows changes in sleep and daily habits at initial visit across age groups. More than half of the patients (51.8%) reported that their current concussion changed their daily habits, with the highest proportion reported among 13–18-year-olds (54.1%, *p* < 0.001) compared to 5–9-year-olds (40.9%) and 10–12-year-olds (45.4%). Approximately 60% of this cohort reported that their sleep changed due to an SRR concussion, with the highest proportion reported among 13–18-year-olds (63.5%) compared to 5–9- and 10–12-year-olds (46.3%, 51.3%, *p* < 0.001). As age increased, the proportion of patients who reported that school made them feel worse increased from 25.6% in 5–9-year-olds and 36.4% in 10–12-year-olds to 41.8% in 13–18-year-olds (*p* < 0.001).

## 4. Discussion

The primary purpose of this study was to describe the demographics, injury mechanisms, initial presentation, and clinical characteristics of SRR concussions among 5–12-year-olds and compare them with adolescents aged 13–18 years. Important differences were identified by age in demographics, medical history, activities during which the concussion was sustained, and the severity and pattern of symptoms. In addition, the impact of the concussion on daily activities, sleep, and school differed by age. These differences are important for the improved identification and diagnosis of concussion, targeted strategies for prevention, as well as management once the injury is sustained. 

When assessing demographic characteristics in pediatric SRR concussions, for ages 5–12 years, boys accounted for a significantly higher proportion (61–70%) of concussions than girls; this is in contrast to 13–18-year-olds, for whom the distribution of sex was more equally divided between boys and girls. Our finding aligns with previous research on SRR concussions indicating that boys account for a larger proportion of concussion cases under the age of 12 years [10] or in elementary and middle school [25] compared to girls. This observation may be due, in part, to sex differences in sports participation, where it has been reported that boys are more physically active than girls even before kindergarten, with a significantly greater proportion participating in sports [26] and having more cumulative total time spent in physical activity [27]. This trend remains throughout childhood [1,28]. In addition to exposure, boys’ sports participation may carry higher risk. Around 76% boys participated in collision or contact sports [29], increasing the risk of head injury. Additionally, maturational differences between boys and girls could also account for the demographic differences observed. At the same chronological age, boys demonstrate less proficiency in fundamental movement skills compared with girls up to 12 years [30]. Lower balance and locomotor skills while playing sports could increase the likelihood that young boys sustain concussion compared to girls. There may also exist implicit gender biases among both parents, clinicians, and other adults who may interact with children in sports contexts in recognizing, diagnosing, and seeking concussion care for girls that may impact these proportions. There is also less access among girls’ sports to specialized athletic training coverage during their sports events compared to boys, which may contribute to lower rates of concussion recognition.

The activities in which SRR concussions were sustained varied by age. Our findings demonstrate that, as the age increased, more SRR concussions occurred in contact or collision sports with the three most common sports being soccer, football, and basketball, which aligns with previously published work [31]. Involvement in contact or collision sports likely increases with age as fundamental movement skills are gradually developing during childhood, leading to more complex movement skills needed by these sports [28]. It has been reported that being struck, kicked, or falling down in football and soccer in adolescents is a common cause of SRR concussions [3,32]. Younger children aged 5–9 years demonstrated greater proportions of concussions sustained during limited- and non-contact sports, as well as recreational and school-based physical activities, such as recess and free play, compared to older children and adolescents, a similar finding to that found in the prior literature [33]. Similarly, previous studies found that elementary- and middle school-aged children were more likely to sustain a concussion from recreational sports compared with high school-aged adolescents [25]. Others reported children aged 6–11 years accounted for 88.6% of concussion cases due to falls from playground equipment [32]. Broadly, SRR concussions in children and adolescents may be prevented through integrating environmental design, sports equipment design, and behavioral modifications to avoid injurious head impact [34,35]. Understanding the age-related differences presented in this study can inform age-specific primary prevention strategies tailored to the unique settings that each age group sustains their injuries. Additionally, recognizing that injuries will still occur and educating parents, school personnel, and health care providers that recreational activities in noncompetitive environments can result in concussion in younger children can help injury identification and facilitate early access to care, thus improving secondary prevention. 

Previous studies assessing age-related differences in concussion symptoms have been inconsistent [36] with some studies reporting younger athletes reporting a greater symptom burden than older athletes [37,38], while other studies demonstrated no age-related differences in symptom presentation and severity [10,39]. However, the majority of these studies included a high proportion of young adults and late adolescents in their samples [12,37]. In our study, we found that younger children aged 5–9 and 10–12 years presented symptoms less frequently, but when they did present them, they reported higher severities among certain somatic symptoms, such as headaches and sensitivity to light, compared to older adolescents. This aligns with prior research that highlighted the importance of somatic symptoms for this age group [16,40] and may be caused by age-related pathophysiological differences [41], or anatomical and physiological vulnerability of young brains [15]. For example, it has been suggested that weaker neck muscles and the larger head-to-head ratio of younger children increases rotational kinematics during events that lead to concussion and nervous system immaturity, including a larger subarachnoid space and incomplete myelination may place younger children at a unique risk of specific concussion symptoms compared to adolescents [41]. Physiological, neurometabolic, and structural brain development likely also plays a role in how concussions may manifest differently across the pediatric age range. Previous evidence also showed that pediatric concussions may lead to altered functional connectivity in pain processing and a disrupted utilization of the vestibular system [42], which may result in a greater severity of headaches, nausea, dizziness, and sensitivity to noise in children. Together, these differences make children more vulnerable to experiencing physical symptoms than adolescents. 

Our findings also show younger children reported a greater severity in irritability and nervousness than adolescents. This may be due to having less experience with brain injury, less concussion knowledge [43], or higher susceptibility to anxiety following SRR concussions [44]. As younger children are less likely to seek professional help from mental health services than adolescents [45], emphasizing the presence of these emotional symptoms and need for specialized mental health care in the younger age groups is an important point for providers and parents to be particularly aware of. 

Drowsiness and fatigue were the highest in those 10–12 years of age, compared to the youngest (5–9 years) and oldest (13–18 years) age groups. In contrast, feeling slowed down was the highest in the adolescents. It is possible that these symptoms represent similar concepts that are conflated in self-reporting or potentially unrecognized by the youngest children. Symptom severity in “slowed down” was significantly higher in adolescents than children, which aligns with previous work [46]. 

Concussion symptoms can interfere with academic performance and the activities of daily living across the pediatric age range [47,48], adversely affecting their quality of life. In our study, more than half of children and adolescents reported overall changes in daily habits due to SRR concussions. The proportion of those reporting changes in daily habits increased as the age increased, with the highest proportion observed in adolescents aged 13–18 years (54.1%) compared to 40.9% of 5–9-year-olds. Sleep was particularly disrupted, with around 60% of adolescents aged 13–18 years affected compared to approximately one-half of those under the age of 12 years. This is consistent with previous studies where adolescent patients more frequently reported sleep problems than children after experiencing concussion [10,16,49]. We found that a greater proportion of adolescents reported that school made them feel worse compared to younger children. Similarly, some studies have shown that excessive school work can also worsen existing symptoms and may even prolong concussion recovery [38,48]. For adolescents, their burden of schoolwork is greater with a higher cognitive demand from learning activities and more social activities in the school environment than younger children. Thus, impaired neurocognitive function as a result of SRR concussions could affect adolescents more, preventing them from achieving their academic goals, thereby leading to more school problems. Academically oriented neurocognitive interventions may be needed to fully support return-to-school for both children and adolescents.

Of note, our study showed no differences in time to presentation to specialty clinic or time in care across age. The prior literature has been mixed on this topic. Howell et al. showed there was no significant age-related difference in time since injury to the first clinical visit [10]. However, another study reported that younger children from elementary/middle school had a significantly delayed time to medical assessment since injury compared to older children in high school [25]. Our specialty-care-based dataset may not reflect the true number of days since injury to initial medical assessment, because children and adolescent patients may have visited another care provider, including an emergency department physician or primary care provider, before presenting to specialty care later. It is also possible that fewer younger patients are referred to specialty care. Regardless, there is clear evidence that delays in initial care negatively affect outcomes; so, efforts should be directed across all age groups to expedite concussion identification and evaluation by health care professionals [50,51,52]. 

There are several limitations to this study. First, the participants in this study were limited to those who visited a specialty care referral program within 28 days of sustaining an SRR concussion. Thus, our results cannot be extrapolated to those who seek care outside of this setting, such as primary care or emergency care, the latter of which may represent a group of more severely injured children. Our results may underestimate the symptom burden for those children. Second, concussion symptom evaluation was based on PCSI, which uses different scales for children and adolescents. We reconciled this discrepancy by normalizing the differing scales in order to compare across age groups, a method that has also been utilized in previously published work [53,54]; however, this approach may add errors to the comparison of symptom burden between the ≤12-year-old age group and those 13 years and older. Third, the cohort was categorized based on chronological age. There is a possibility that children and adolescents in different stages of maturity were assigned to the same age group; thus, maturity-associated factors (e.g., Tanner stage) may influence variations in symptom perception and reporting. The direction of the effect of this limitation is challenging to predict. Lastly, the participants were predominantly White. This limitation warrants further study to examine larger representative samples to understand the effect of racial and ethnic diversity on our findings. 

## 5. Conclusions

Among SRR-concussed youths at an initial visit to specialty care, developmental changes with age lead to variability in the mechanism of SRR concussions, symptom profile, and clinical outcomes. Children aged ≤12 years sustaining SRR concussions were more likely to be male and to have sustained their concussion from limited- and non-contact sports as well as free play and school physical activities than older adolescents. Thus, prevention strategies, environmental design, and concussion education should incorporate these differences to ensure safety for all children in sports and recreational activities. Children aged ≤ 12 years demonstrated lower rates of presenting most symptoms, but reported a higher severity in certain somatic, emotional, and fatigue symptoms than older adolescents. As the age increased, the patients reported a greater impact of concussions on the overall daily habits, sleep, and schoolwork. These results suggest that different ages experience unique challenges following concussions that may be developmental in nature. Future research should explore the results of this study in diverse populations or other health care settings. There is an urgent need for an additional study of SRR concussions across the pediatric age range in order to improve the pediatric-centered management of concussions.

## Figures and Tables

**Table 1:** Demographic characteristics of patients aged 5–18 years presenting with an SRR concussion.

Variables	All (*n* = 3280)		5–9 Years (*n* = 164)		10–12 Years (*n* = 604)		13–18 Years (*n* = 2512)		*p*-Values
	*n*	%	*n*	%	*n*	%	*n*	%	
**Sex**									<0.001 ^b,c^
Female	1525	46.5	48	29.3	233	38.6	1244	49.5	
Male	1755	53.5	116	70.7	371	61.4	1268	50.5	
**Race/ethnicity**									0.08
Non-Hispanic White	2319	70.7	119	72.6	405	67.1	1795	71.5	
Non-Hispanic Black	331	10.1	20	12.2	59	9.8	252	10.0	
Non-Hispanic other	413	12.6	19	11.6	93	15.4	301	12.0	
Hispanic or Latino	198	6.0	5	3.0	40	6.6	153	6.1	
Unknown	19	0.6	1	0.6	7	1.2	11	0.4	
**Concussion history**									<0.001 ^a,b,c^
Yes	1271	38.8	23	14.0	185	30.6	1063	42.3	
No	1998	60.9	141	86.0	416	68.9	1441	57.4	
Unknown	11	0.3	0	0.0	3	0.5	8	0.3	
**Time in care**									0.215
≤28 days	2251	68.6	121	73.8	422	69.9	1708	68.0	
>28 days	1029	31.4	43	26.2	182	30.1	804	32.0	
	**median**	**IQR**	**median**	**IQR**	**median**	**IQR**	**median**	**IQR**	
**Days from injury to initial clinic visit**	11	6–17	12	7–17	11	6–17	11	6–17	0.563
**Total days in care**	14	0–39	14	0–29.8	14	0–40.2	14	6–39	0.086
**Height (cm)**	164.5	156.5–172.5	133.9	128.8–140.3	151.0	145.3–158.3	167.7	161.4–174.6	<0.01 ^a,b,c^
**Weight (kg)**	59.0	49.0–69.7	30.4	26.6–35.6	43.6	37.2–52.0	62.7	55.2–72.9	<0.01 ^a,b,c^
**BMI (kg/m^2^)**	21.5	19.2–24.2	16.9	15.7–18.5	18.8	16.9–21.6	22.1	20.2–24.8	<0.01 ^a,b,c^

Note: ^a^ denotes a significant difference was detected between 5–9- and 10–12-year-olds; ^b^ denotes a significant difference was detected between 10–12- and 13–18-year-olds; and ^c^ denotes a significant difference was detected between 5–9- and 13–18-year-olds. IQR denotes interquartile range. BMI denotes body mass index.

**Table 2:** Classification of activities in which an SRR concussion was sustained by level of contact among 5–18 year-olds.

Sport Types	All (*n* = 3280)		5–9 Years (*n* = 164)		10–12 Years (*n* = 604)		13–18 Years (*n* = 2512)	
	*n*	%	*n*	%	*n*	%	*n*	%
**Contact or collision sports**	**2304**	**70.2**	**96**	**58.5**	**435**	**72.0**	**1773**	**70.6**
Basketball	365	11.1	18	11.0	78	12.9	269	10.7
Field hockey	88	2.7	4	2.4	10	1.7	74	2.9
Football	505	15.4	27	16.5	103	17.1	375	14.9
Ice hockey	240	7.3	13	7.9	51	8.4	176	7.0
Lacrosse	198	6.0	2	1.2	20	3.3	176	7.0
Martial arts	18	0.5	1	0.6	2	0.3	15	0.6
Rugby	58	1.8	0	0.0	1	0.2	57	2.3
Soccer	652	19.9	28	17.1	149	24.7	475	18.9
Water polo	20	0.6	0	0.0	1	0.2	19	0.8
Wrestling	140	4.3	3	1.8	19	3.1	118	4.7
Other	20	0.6	0	0.0	1	0.2	19	0.8
**Limited contact sports**	**838**	**25.5**	**47**	**28.7**	**144**	**23.8**	**647**	**25.8**
Baseball	75	2.3	9	5.5	19	3.1	47	1.9
Biking	35	1.1	4	2.4	5	0.8	26	1.0
Cheerleading	164	5.0	4	2.4	24	4.0	136	5.4
Dodgeball	15	0.5	0	0.0	4	0.7	11	0.4
Flag football	25	0.8	2	1.2	7	1.2	16	0.6
Gymnastics	53	1.6	2	1.2	11	1.8	40	1.6
Horseback riding	40	1.2	2	1.2	9	1.5	29	1.2
Ice skating	20	0.6	5	3.0	6	1.0	9	0.4
Kickball	11	0.3	3	1.8	7	1.2	1	0.0
Roller skating	6	0.2	1	0.6	1	0.2	4	0.2
Skateboarding	13	0.4	0	0.0	0	0.0	13	0.5
Skiing	57	1.7	7	4.3	11	1.8	39	1.6
Snowboarding	75	2.3	2	1.2	9	1.5	64	2.5
Softball	98	3.0	1	0.6	11	1.8	86	3.4
Trampoline	6	0.2	1	0.6	2	0.3	3	0.1
Volleyball	101	3.1	1	0.6	6	1.0	94	3.7
Weightlifting	7	0.2	0	0.0	0	0.0	7	0.3
Other	37	1.1	3	1.8	12	2.0	22	0.9
**Non-contact sports**	**103**	**3.1**	**13**	**7.9**	**13**	**2.2**	**77**	**3.1**
Climbing	4	0.1	2	1.2	1	0.2	1	0.0
Color guard	13	0.4	0	0.0	0	0.0	13	0.5
Crew	7	0.2	0	0.0	0	0.0	7	0.3
Dancing	16	0.5	2	1.2	1	0.2	13	0.5
Swimming	36	1.1	6	3.7	3	0.5	27	1.1
Tennis	4	0.1	0	0.0	0	0.0	4	0.2
Track and field	8	0.2	0	0.0	0	0.0	8	0.3
Other	15	0.5	3	1.8	8	1.3	4	0.2
**Other activities**	**35**	**1.1**	**8**	**4.9**	**12**	**2.0**	**15**	**0.6**
Fitness training	4	0.1	0	0.0	0	0.0	4	0.2
Physical education	9	0.3	3	1.8	1	0.2	5	0.2
Free playing	13	0.4	3	1.8	8	1.3	2	0.1
Recess	3	0.1	1	0.6	2	0.3	0	0.0
Other	6	0.2	1	0.6	1	0.2	4	0.2

**Table 3:** Comparison of PCSI symptom presence by age group.

Symptoms	5–9 Years		10–12 Years		13–18 Years		*p*-Values
	*n*	%	*n*	%	*n*	%	
Headaches	120	78.4	447	82.5	2115	86.0	0.007 ^c^
Nausea	63	41.2	219	40.5	1049	42.7	0.627
Light	67	55.4	348	64.3	1800	73.3	<0.001 ^b,c^
Noise	66	54.5	289	53.4	1472	59.9	0.014 ^b^
Dizziness	59	38.6	299	55.2	1506	61.2	<0.001 ^a,b,c^
Balance	35	28.9	214	39.6	1243	50.5	<0.001 ^b,c^
Vision	31	25.6	165	30.5	1014	41.3	<0.001 ^b,c^
Difficulty in concentrating	80	52.3	324	59.9	1813	73.8	<0.001 ^b,c^
Difficulty in remembering	42	34.7	218	40.3	1292	52.6	<0.001 ^b,c^
Slowed down	62	51.2	330	61.0	1512	61.5	0.076
Mentally foggy	48	39.7	282	52.1	1607	65.4	<0.001 ^b,c^
Drowsiness	64	52.9	346	64.0	1533	62.3	0.075
Fatigue	68	56.2	351	64.9	1710	69.5	0.002 ^c^
Irritability	74	48.4	272	50.3	1352	55.0	0.050
Sadness	38	31.4	145	26.8	795	32.4	0.042 ^b^
Nervousness	42	34.7	196	36.2	802	32.6	0.263

Note: ^a^ denotes a significant difference was detected between 5–9- and 10–12-year-olds; ^b^ denotes a significant difference was detected between 10–12- and 13–18-year-olds; and ^c^ denotes a significant difference was detected between 5–9- and 13–18-year-olds.

**Table 4:** Comparison of normalized PCSI symptom severity scores by age group.

Symptoms	5–9 Years		10–12 Years		13–18 Years		*p*-Values
	Mean	SD	Mean	SD	Mean	SD	
Headaches	52.6	34.8	59.4	35.5	47.5	28.4	<0.001 ^a,b^
Nausea	24.5	32.0	24.2	32.0	18.0	25.2	<0.001 ^b,c^
Light	34.7	35.3	45.4	39.2	39.0	32.4	<0.001 ^a,b^
Noise	37.2	38.5	36.2	38.1	29.4	30.7	<0.001 ^b,c^
Dizziness	21.2	28.5	33.4	33.7	27.7	28.3	<0.001 ^a,b,c^
Balance	18.2	31.0	23.1	30.9	20.8	25.4	0.089
Vision	14.5	26.2	18.2	29.6	17.6	26.0	0.377
Difficulty in concentrating	30.4	32.1	39.9	37.5	40.3	32.9	0.002 ^a,c^
Difficulty in remembering	18.2	25.8	23.8	31.5	24.3	29.5	0.084
Slowed down	21.5	26.7	28.9	29.9	30.6	30.9	0.004 ^a,c^
Mentally foggy	24.0	32.3	33.3	35.8	33.2	31.9	0.009 ^a,c^
Drowsiness	33.5	35.6	45.8	39.7	31.8	31.6	<0.001 ^a,b^
Fatigue	35.5	35.6	46.1	39.4	37.5	32.5	<0.001 ^a,b^
Irritability	32.0	37.0	33.5	37.3	27.2	31.3	<0.001 ^b^
Sadness	18.6	29.7	16.2	28.7	13.6	24.0	0.016
Nervousness	21.9	32.8	22.2	32.0	14.1	24.4	<0.001 ^b,c^

Note: ^a^ denotes a significant difference was detected between 5–9- and 10–12-year-olds; ^b^ denotes a significant difference was detected between 10–12- and 13–18-year-olds; and ^c^ denotes a significant difference was detected between 5–9- and 13–18-year-olds. SD denotes standard deviation.

**Table 5:** Frequencies of clinical characteristics by age group.

	All (*n* = 3280)		5–9 Years (*n* = 164)		10–12 Years (*n* = 604)		13–18 Years (*n* = 2512)		*p*-Values
	*n*	%	*n*	%	*n*	%	*n*	%	
**Daily habits changes**									<0.001 ^b,c^
Yes	1700	51.8	67	40.9	274	45.4	1359	54.1	
No	1413	43.1	89	54.3	294	48.7	1030	41.0	
Not reported	167	5.1	8	4.9	36	6.0	123	4.9	
**Sleep changes**									<0.001 ^b,c^
Yes	1980	60.4	76	46.3	310	51.3	1594	63.5	
No	1192	36.3	85	51.8	267	44.2	840	33.4	
Not reported	108	3.3	3	1.8	27	4.5	78	3.1	

Note: ^b^ denotes a significant difference was detected between 10–12- and 13–18-year-olds; and ^c^ denotes a significant difference was detected between 5–9- and 13–18-year-olds. Daily habits changes included lightheadedness when standing up, increased/decreased appetite, increased/decreased urination, urination accidents, constipation, and diarrhea.

## Data Availability

The data presented in this study are available upon request from the author KBA, subject to data use agreements between the parties.

## Supplementary Materials

The following supporting information can be downloaded at: https://www.mdpi.com/article/10.3390/diagnostics14182042/s1. Table S1: Pre-existing medical conditions among concussed patients aged 5–18 years at initial SRR concussion visit.
