# Supplementary material for: Age-Related Variations in Clinical Profiles for Children with Sports- and Recreation-Related Concussions
Source: Diagnostics (Basel). 2024 Sep 14;14(18):2042. doi: 10.3390/diagnostics14182042 (PMC11431309; doi:10.3390/diagnostics14182042)
Supplement: Supplementary file 1 [file diagnostics-14-02042-s001.zip › diagnostics-3183733-supplementary.pdf]

Supplementary Table S1. Pre-existing medical conditions among concussed patients aged 5–18 years at initial SRR concussion visit.

	**All** (*n*= 3280)		**5-9 years** (*n*=164)		**10-12 years** (*n*=604)		**13-18 years** (*n*=2512)		***p*-Values**
	n	**%**	***n***	**%**	***n***	**%**	***n***	**%**	
**Learning and/or Developmental**									
ADHD/ADD	449	13.69	13	7.93	81	13.41	355	14.13	0.073
Learning disability	317	9.66	11	6.71	54	8.94	252	10.03	0.298
Dyslexia	102	3.11	2	1.22	22	3.64	78	3.11	0.271
IEP or 504 plan	623	18.99	23	14.02	132	21.85	468	18.63	0.037
Autism	38	1.16	2	1.22	8	1.32	28	1.11	0.902
**Mood-related**									
Anxiety	611	18.63	18	10.98	100	16.56	493	19.63	0.007[c]
Bipolar disorder	22	0.67	0	0.00	5	0.83	17	0.68	0.507
Depression	306	9.33	2	1.22	28	4.64	276	10.99	< 0.001[b,c]
Other psychiatric disorders	64	1.95	2	1.22	16	2.65	46	1.83	0.32
**Visual/Vestibular**									
Eye-patching	66	2.01	3	1.83	18	2.98	45	1.79	0.163
Amblyopia	88	2.68	6	3.66	20	3.31	62	2.47	0.372
Strabismus	63	1.92	5	3.05	22	3.64	36	1.43	< 0.001[b]
Strabismus eye surgery	37	1.13	4	2.44	13	2.15	20	0.80	0.005[b]
Glasses or contacts for distance	980	29.88	23	14.02	134	22.19	823	32.76	< 0.001[b,c]
Glasses or contacts for reading	588	17.93	21	12.80	113	18.71	454	18.07	0.174
**Physical**									
Chronic headaches	244	7.44	1	0.61	40	6.62	203	8.08	0.001[a,c]
Migraines	360	10.98	3	1.83	52	8.61	305	12.14	< 0.001[a,c]
Motion sickness	404	12.32	20	12.20	79	13.08	305	12.14	0.783
Postural Orthostatic Tachycardia Syndrome	24	0.73	1	0.61	3	0.50	20	0.80	0.732
Seizures/Epilepsy	37	1.13	4	2.44	5	0.83	28	1.11	0.231
Sleep problems	276	8.41	9	5.49	50	8.28	217	8.64	0.353
Tic disorder	47	1.43	4	2.44	6	0.99	37	1.47	0.377
**Therapy**									
Reading therapy	105	3.20	9	5.49	21	3.48	75	2.99	0.200
Speech therapy	287	8.75	27	16.46	70	11.59	190	7.56	< 0.001[b,c]
Vestibular therapy	32	0.98	2	1.22	5	0.83	25	1.00	0.893
Vision therapy	99	3.02	5	3.05	25	4.14	69	2.75	0.188

Note: [a] denotes a significant difference was detected between 5–9- and 10–12-year-olds; [b] denotes a significant difference was detected between 10–12- and 13–18-year-olds; and [c] denotes a significant difference was detected between 5–9- and 13–18-year-olds.
